# Cucurbitacin E Inhibits Huh7 Hepatoma Carcinoma Cell Proliferation and Metastasis via Suppressing MAPKs and JAK/STAT3 Pathways

**DOI:** 10.3390/molecules25030560

**Published:** 2020-01-28

**Authors:** Yang Liu, Heng Yang, Qiang Guo, Tingting Liu, Yong Jiang, Mingbo Zhao, Kewu Zeng, Pengfei Tu

**Affiliations:** State Key Laboratory of Natural and Biomimetic Drugs, School of Pharmaceutical Sciences, Peking University, Beijing 100191, China; 1311210122@bjmu.edu.cn (Y.L.); 15111322446@163.com (H.Y.); qiangguo@pku.edu.cn (Q.G.); cpultt126@126.com (T.L.); yongjiang@bjmu.edu.cn (Y.J.); zmb@bjmu.edu.cn (M.Z.)

**Keywords:** Cucurbitacin E, hepatocellular carcinoma, proliferation, migration, MAPK pathway, JAK/STAT3 pathway

## Abstract

Cucurbitacin E (CuE), a highly oxygenated tetracyclic triterpene from Cucurbitaceae, has shown to exhibit potent cytotoxic and anti-proliferative properties against several human cancer cells. However, the underlying effects and mechanisms of CuE regarding hepatocellular carcinoma (HCC) have not been well understood. In the current study, unbiased RNA-sequencing (RNA-seq) and bioinformatics analysis was applied to elucidate the underlying molecular mechanism. CuE could significantly inhibit cell proliferation and migration of Huh7 cells, meanwhile CuE exhibited potent anti-angiogenic activity. RNA-seq analysis revealed that CuE negatively regulated 241 differentially expressed genes (DEGs) involved in multiple processes including cytoskeleton formation, angiogenesis and focal adhesion. Further analysis revealed that CuE effectually regulated diversified pharmacological signaling pathways such as MAPKs and JAK-STAT3. Our findings demonstrated the role of CuE in inhibiting proliferation and migration, providing an insight into the regulation of multiple signaling pathways as a new paradigm for anti-cancer treatment strategy.

## 1. Introduction

Hepatocellular carcinoma (HCC) is reported to be the most common cancer and aggressive human malignancies worldwide [1,2,3]. High metastasis and invasion of HCC remains the greatest challenge in clinical management. However, only less than thirty percent of patients meet the surgical requirements [4]. Obviously, most of the patient mortality is attributed to high recurrence rate and extensive metastasis in liver cancer treatment [5].

Cancer development is a multi-step process that arises from a series of genetic and epigenetic events leading to multiple alterations in signaling pathways, such as the growth factor pathway, VEGF pathway, MAPK, and JAK/STAT pathway [6,7]. Targeting these signaling pathways has been considered a promising strategy for cancer therapy. Single target interventions are largely ineffective and commonly drug resistance for complex cancer therapy [8,9]. Therefore, it is difficult to achieve the desired therapeutic effect by using single target therapy. For these situations, discovery of molecules that target multiple proteins or signaling pathways involved in HCC may be a more effective therapeutic strategy [10,11,12].

Natural products, the most important chemical library, have long been playing significant roles in contributing to the discovery and development of new drugs, such as vincristine, cytarabine, doxorubicin, paclitaxel, and docetaxel [13,14]. Therefore, it has become a promising cut-in point to explore new drugs derived from natural products against cancer. Cucurbitacins are a family of natural products isolated from Cucurbitaceae plants, and possess a wide spectrum of pharmacological effects including anti-cancer, anti-diabetic, cytoprotective, and anti-inflammatory activities [15,16,17,18]. Cucurbitacin E (CuE), a highly oxygenated triterpene of cucurbitacins, is known to have a variety of pharmacological effects such as hepatoprotective, anti-inflammatory and anti-cancer activities [19,20,21,22,23,24]. Previous investigations have shown that CuE inhibits cell proliferation, migration, and invasion through different molecular mechanisms in vitro and in vivo, indicating that CuE exerts prominent investigation prospect in the treatment of various cancers. However, the function of CuE on HCC migration and its related molecular mechanism have not yet been investigated.

In this study, we found that CuE could significantly inhibit the proliferation and migration of Huh7 cells by disrupting cytoskeleton depolymerization and inducing cell cycle arrest. Subsequently RNA-sequencing (RNA-seq) and bioinformatics analysis revealed multiple processes that are involved in cell proliferation and migration. Further study revealed that CuE inhibited Huh7 proliferation and metastasis via simultaneously regulating diversified pharmacological targets, including MAPKs and JAK/STAT. Taken together, our findings show that CuE is a promising drug candidate for inhibiting cancer cell proliferation and migration, which suggests a crucial therapeutic strategy by targeting multiple signaling pathways for HCC.

## 2. Results

### 2.1. CuE Inhibited the Proliferation, Migration and Angiogenesis of Huh7 Cells

**Figure 1:**
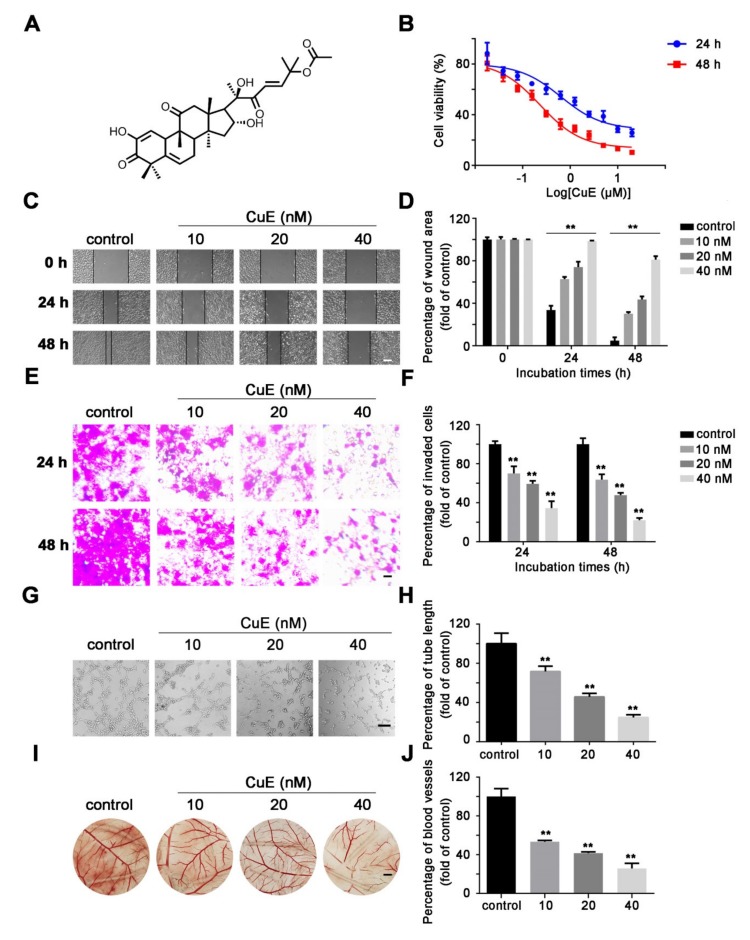
CuE inhibited migration, invasion and angiogenesis. (**A**) Chemical structure of CuE. (**B**) Huh7 cells were treated with various concentrations of CuE for 24 or 48 h and the cell viability was evaluated by MTT assay. (**C**) Wound healing assay. HuH7 cells were treated with CuE (0, 10, 20, and 40 nM) for 24 h and 48 h, and the migration of Huh7 cells was quantified by measuring wound areas (bar = 200 μm). (**D**) Relative migration was calculated by comparing the cells migrated to the wounded area after CuE treatment, with those of control cells. Data are mean ± SD of three independent experiments (n = 3). **: p < 0.01, compared with the control group. (**E**) The cell invasion was determined by transwell invasion assay. After incubation at 37 °C for 24 and 48 h, the migrated cells were fixed and stained with crystal violet solution (bar = 400 μm). (**F**) Relative migration was calculated by comparing the cells migrated through the chamber membrane after CuE treatment, with those of control cells. Data are mean ± SD of three independent experiments (n = 3). **: p < 0.01, compared with the control group. (**G**) Inhibitory effect of CuE on the spontaneous tube formation of HUVEC cells. HUVECs were grown on thin matrigel layers for 6 h in the presence of CuE (0, 10, 20, and 40 nM); images shown are representative of three independent experiments (bar = 200 μm). (**H**) Calculations were based on the length of the tubes measured using ImageJ. Data are mean ± SD of three independent experiments (n = 3). **: p < 0.01, compared with the control group. (**I**) The antiangiogenic activity of CuE in chorioallantoic membranes (CAMs). Coverslips loaded with vehicle or CuE was applied to the CAM surface for 72 h (bar = 200 mm). (**J**) Calculations were based on the ration of inhibited eggs relative to the total of number of live eggs. Data are mean ± SD of three independent experiments (n = 3). **: p < 0.01, compared with the control group.

The anti-proliferative effect of CuE on Huh7 cells was evaluated by an MTT assay. CuE significantly inhibited cell proliferation in a concentration- and time-dependent manner (Figure 1B). Next, we detected the effect of CuE on cell migration and invasion. Wound healing assay showed that CuE could remarkably reduce migratory Huh7 cells (Figure 1C,D). Moreover, we also tested the effect of CuE on the invasive capability by transwell invasion assays. Results showed that CuE was capable of blocking Huh7 cell invasion (Figure 1E,F). Furthermore, anti-angiogenic activity of CuE demonstrated that CuE decreased tube formation in HUVECs and inhibited neovascularization in CAM assays, respectively (Figure 1G–J). The above findings suggested that CuE exhibited obvious anti-migrative, anti-invasive, and anti-angiogenesis effects, indicating a potential novel therapeutic candidate for the treatment of cancer metastasis.

### 2.2. Bioinformatics Analysis for the CuE-Regulated Signaling Networks

**Figure 2:**
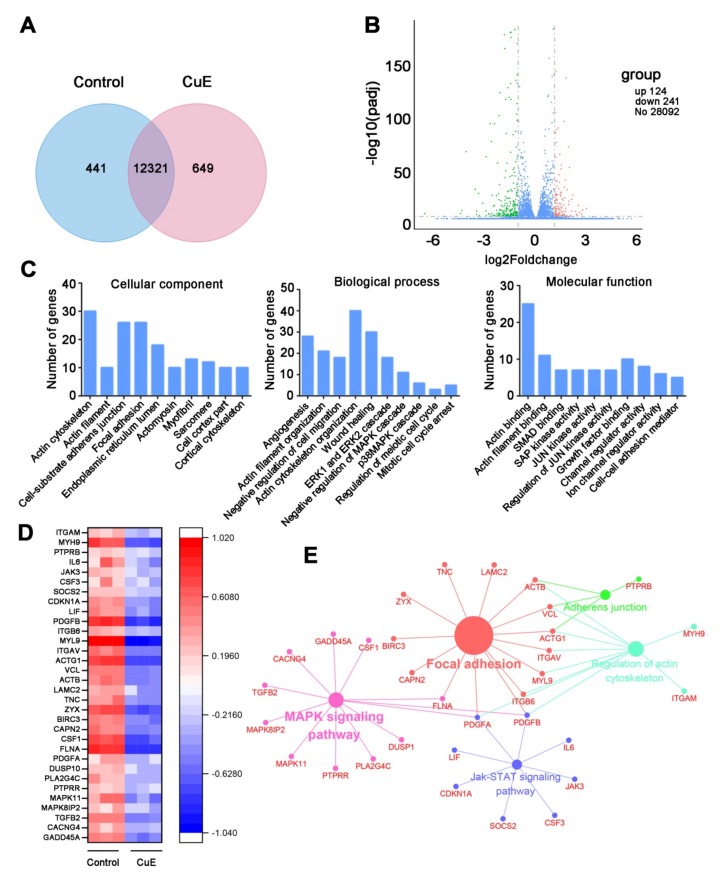
RNA-seq analysis for CuE-altered genes. Cells were treated with vehicle, or 40 nM of CuE for 24 h, respectively. (**A**) Venn diagram showing numerical distribution of differentially expressed genes in HUh7 cells. (**B**) The volcano of differentially expressed genes. (**C**) Genes significantly down-regulated by CuE were classified into different CCs, BPs, and MFs. (**D**) Heat map of down-regulated genes involved in significantly canonical pathways after treatment with CuE in Huh7 cells. (**E**) KEGG pathway enrichment analysis for the significantly down-regulated gene predicted the significantly canonical pathways.

We profiled the effects of CuE on gene expression in Huh7 cells after 24 h treatment. The relationship of detected genes in the two groups was described in a Venn diagram (Figure 2A). A total of 365 DEGs showed significant alteration at p < 0.05 with a fold change > 2, of which 124 genes were markedly up-regulated while 241 genes were down-regulated. The volcano plot revealed the up-regulated and down-regulated genes in each dataset (Figure 2B). To elucidate the potential functions of these DEGs, we performed GO enrichment analysis, including cellular components (CCs), biological processes (BPs), and molecular functions (MFs). As shown in Figure 2C, for BPs, GO terms were significantly enriched in actin cytoskeleton, cell-substrate adherens junction, focal adhesion, and endoplasmic reticulum lumen. MFs analysis showed that the DEGs were particularly enriched in actin cytoskeleton organization, wounding healing, angiogenesis, MAPK, and cell cycle arrest. Similarly, GO terms enriched in actin binding, actin filament binding, and growth factor binding.

To further visualize CuE regulated pharmacological network, the 241 CuE-negatively regulated genes were examined for enrichment in KEGG pathway database from ClueGO plug-in. The global pathway network depicting KEGG pathway analysis were mainly involved in focal adhesion, MAPK, JAK-STAT, and regulation of actin cytoskeleton, which were mapped in Figure 2D–E. Together, these findings suggested that CuE could effectively regulate multiple processes and cellular signaling pathways, and thereby exerted its anti-proliferative and anti-metastatic effects.

**Figure 3:**
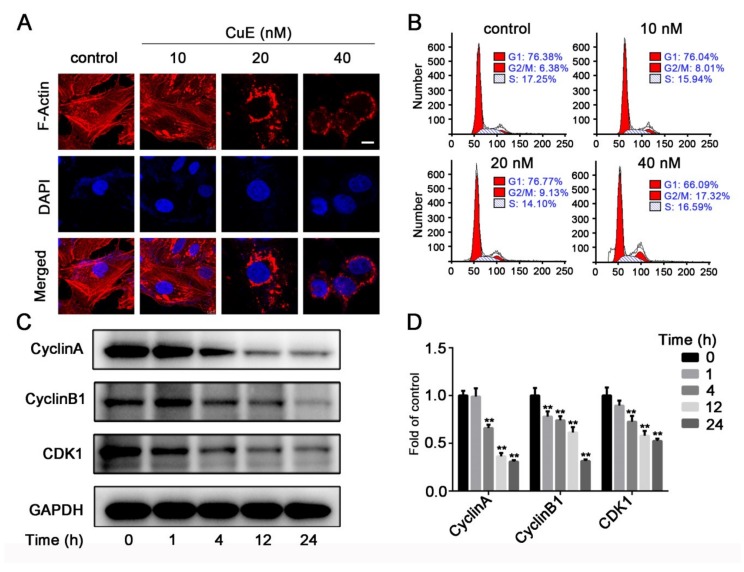
(**A**) CuE effected cytoskeletal organization. Huh7 cells grown coverslips were treated with CuE (0, 10, 20, and 40 nM) for 6 h. Immunocytochemistry was conducted using rhodamine-conjugated phalloidin to visualize F-actin fibers (bar = 10 μm). (**B**) Huh7 cells were treated with CuE (0, 10, 20, and 40 nM) for 24 h. The cells were fixed and stained with propidium iodide (PI), and the DNA content was analyzed by flow cytometry. (**C**) Huh7 cells were treated with CuE (40 nM) for different time points (0, 1, 4, 12, and 24 h), and the protein levels of cyclin A, cyclin B1 and CDK1 were detected by Western blotting. (**D**) Quantitative analysis for relative protein expression levels of CyclinA, CyclinB1, and CDK1 was performed by normalizing to GAPDH. Data are mean ± SD of three independent experiments (n = 3). **: p < 0.01.

The cytoskeleton plays an important role in maintaining cell morphology and migration, and thus we hypothesized that CuE affects migration by affecting cytoskeletal proteins. To evaluate the effect on cytoskeleton, F-actin was stained with rhodamine-conjugated phalloidin and then observed by confocal microscopy (Figure 3A). The microfilaments of untreated tumor cells showed intact filaments, while the microfilaments of the CuE-treated group were broken and aggregated. Therefore, we confirmed that CuE could significantly inhibit cell migration by destroying cytoskeletal organization.

To assess the effect of CuE on cell cycle progression, cell cycle distribution analysis of Huh7 cells was carried out using flow cytometry. As shown in Figure 3B, treatment with different concentrations of CuE caused a dose-dependent increase of the cell population in the G_2_/M phase, which may imply that the Huh7 cells underwent cell cycle arrest. Our results indicate that treatment with CuE increased the cell populations in the G_2_/M phase, while simultaneously reducing the number of cells in the S phase.

Cell cycle progression is tightly regulated through a complex network of positive and negative cell cycle regulatory molecules, such as cyclin-dependent kinases (CDKs) and cyclins. To elucidate the specific cell cycle regulatory proteins responsible for the cell cycle arrest mediated by CuE in Huh7 cells, the effect on cell cycle related proteins involved in G_2_/M transition including cyclin A, cyclin B1 and CDK1, was analyzed by Western blot. Consequently, we found that CuE treatment caused a marked decrease in cyclin A, cyclin B1, and CDK1 protein expression (Figure 3C). These results imply that the growth inhibition by Huh7 cells may target several components of the cell cycle regulatory apparatus. The above results suggested that CuE arrested cell cycle at the G_2_/M phase through regulating cyclin A, cyclin B1, and CDK1.

### 2.3. CuE Inhibited JNK/ERK/p38 MAPK Pathways in Huh7 Cells

**Figure 4:**
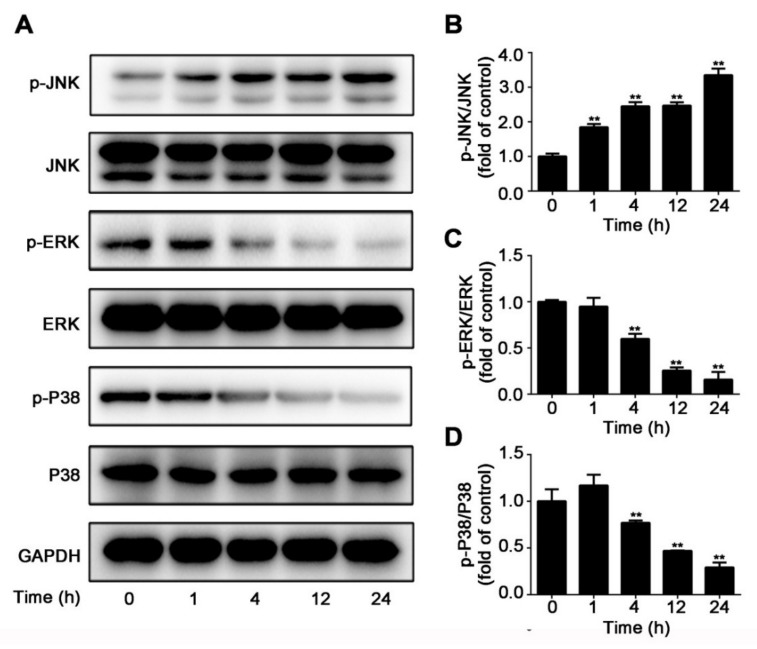
CuE regulated JNK, ERK, and p38 MAPKs in Huh7 cells. Cells were treated with CuE (40 nM) for various time (0, 1, 4, 12, and 24 h). (**A**) Phosphorylations of JNK, ERK, and p38 protein were determined by Western blot assay. (**B**–**D**) Quantitative analysis for relative phosphorylation levels of JNK (**B**), ERK (**C**), and p38 MAPK (**D**) was performed by normalizing to the control group. Data are mean ± SD of three independent experiments (n = 3). **: p < 0.01, compared with the control group.

The initiation of mitogen-activated protein kinase (MAPK)/ERK pathway is a major pathway regulating cell survival, proliferation, and metastasis. In an attempt to validate the obtained suggestions, Western blotting was conducted to detect a number of candidates. To further characterize the mechanism underlying the inhibition of cell proliferation by CuE, we checked the effect of CuE on representative key protein expression (JNK, ERK, and p38) of these pathways. As shown in Figure 4A–D, while CuE suppressed the phosphorylation of ERK and p38, it increased JNK expression compared with the vehicle control, which suggested that CuE suppressed Huh7 cell migration and invasion by positive regulation JNK pathway and negative regulating ERK/p38 pathway. The above results suggested that CuE inhibited Huh7 cells proliferation, migration and invasion. Such effects might be related to the regulation of the MAPK pathway.

### 2.4. CuE Inhibited JAK3/STAT3 Signaling

**Figure 5:**
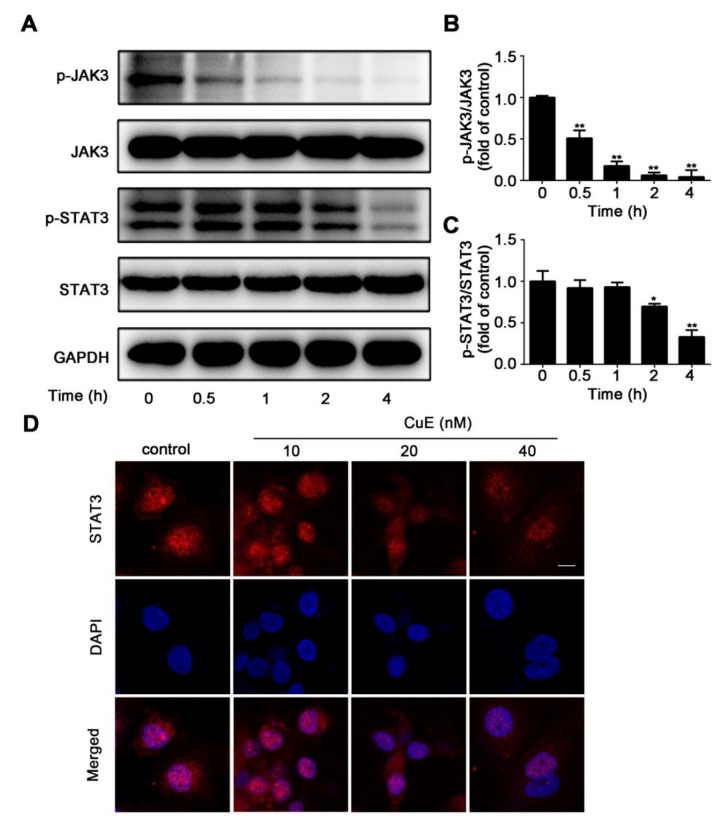
CuE restrained the activation of JAK3 and STAT3 in Huh7 cells. Cells were treated with CuE (40 nM) for various hours. (**A**) Phosphorylations of JAK3 and STAT3 protein were determined by Western blotting. (**B**-**C**) Quantitative analysis for relative phosphorylation levels of JAK3 (**B**) and STAT3 (**C**) was performed by normalizing to the control group. Data are mean ± SD of three independent experiments (n = 3). *: p < 0.05, **: p < 0.01, compared with the control group. (**D**) Effect of CuE on nuclear translocation of p-STAT3 in Huh7 cells. Immunofluorescent analysis was conducted with antibody of p-STAT3 and secondary antibody conjugated to rabbit Dylight-594. Images were captured using a confocal laser scanning microscope.

The JAK/STAT-mediated signaling cascade represents essential roles for proliferation or differentiation, and development. Recent studies showed that persistently activated JAK/STAT signaling correlates with tumorigenesis and cancer progression through its intimate connection to growth factor signaling and observed high frequency in human cancers. As shown in Figure 5A, CuE induced a time-dependent decrease in the level of p-JAK3 and p-STAT3. No detectable changes in the JAK3 and STAT3 were seen. Figure 5B,C also show densitometric analysis of Western blot bands. There was a gradual decrease in p-JAK3/JAK3 and p-STAT3/STAT3 ratios with increasing the concentration of CuE. Immunofluorescent analysis was also conducted to evaluate the effect of CuE on nuclear translocation of p-STAT3. As shown in Figure 5B, CuE attenuated the phosphorylation of STAT3. Consistently, CuE effectively blocked constitutive STAT3 phosphorylation as well as its nuclear translocation (Figure 5C,D). The decrease in cell viability can be attributable to the significant increase in apoptotic cell death and the occurrence of cell cycle arrest after inhibition of the JAK/STAT3 signaling pathway.

## 3. Discussion

Despite numerous advances in cancer treatments, distant metastasis remains the most challenging leading to death in patients with HCC. Cancer metastasis is known to be a complex process that involves multiple steps including migration, invasion and adhesion. Metastasis and invasion of cancer cells and lack of effective treatment have promoted a great amount of novel anti-cancer drugs derived from natural products. CuE, a critical member of cucurbitacin family, exhibits a cytotoxicity effect in several cancers, such as cervical cancer, osteosarcoma, nasopharyngeal carcinoma, and lung cancer. However, little research has been focused on proliferation and metastasis of HCC to delineate underlying effects of CuE. Thus, a comprehensive understanding of the mechanisms of CuE is critical for developing novel treatment strategies for HCC. In this study, we found CuE could significantly inhibit Huh7 cell viability. Further investigation demonstrated that CuE could significantly inhibit Huh7 metastasis and invasion, and drastically suppress neovascularization and tube formation. Further studies into possible mechanisms revealed that CuE inhibited Huh7 migration and invasion via suppressing MAPKs and JAK/STAT3 signaling pathway, together with the regulation of cell cycle arrest and cytoskeleton.

RNA-seq is a powerful approach for investigating drug-related gene expression alterations [25,26]. RNA-seq can determine global transcriptional effects of drugs and significantly accelerate the efficiency and success rate of drug discovery. So far, RNA-seq has been successfully applied to target identification, pharmacological mechanisms, and drug resistance studies for numerous bioactive molecules such as polyphenone, schisandrin B, and oldenlandia diffusa [27,28]. Since CuE exhibited potent inhibitory effect on the proliferation, migration, invasion, and anti-angiogenic activity, RNA-seq was used to investigate its underlying molecular mechanism. Bioinformatics analysis results revealed that several critical signaling pathways were dramatically suppressed, especially on the regulation of the actin cytoskeleton involved in multiple complicated CCs, BPs, and MFs. Actin microfilaments (F-actin) are crucial components of the cytoskeleton, which is responsible for the cell morphology and movement of eukaryotic cells [29,30]. During the movement of cells, the polymerization of actin monomers into polarized F-actin plays a pivotal role, and regulation of this step is a promising strategy in anti-tumor treatment [31,32]. F-actin staining assays clearly demonstrated the aberrant bundling and accumulation of F-actin, which can be attributed to inhibiting its depolymerisation effect by CuE.

The MAPK pathway is an important signaling pathway involved in cell proliferation, differentiation, migration, and apoptosis [33,34,35]. In mammalians, MAPK contain three individual molecules JNK, ERK, and p38 kinase. Hyper-activation of MAPK signaling pathway has been known to frequently occur in many human cancers. In the present study, CuE inhibited Huh7 proliferation and migration by increasing JNK activation and inhibiting ERK activation in a time-dependent manner, which was consistent with its effect on triple-negative breast cancer cells. Besides, our results revealed that p38 phosphorylation was also suppressed by CuE treatment. Therefore, we speculated that CuE exerted its anti-migratory effect by increasing JNK activation as well as suppressing ERK and p38 activation.

STAT3 is one of the most important members of the STAT family and is closely associated with cell proliferation and angiogenesis. Importantly, STAT3 activation has been documented in several tumor types and is associated with tumorigenesis. Moreover, it is considered to be an oncogene and a promising target for HCC. Therefore, aberrant signaling of the JAK/STAT3 activation represents a potential therapeutic strategy for HCC [36,37]. In our investigation, we found that CuE significantly suppressed JAK2 and STAT3 phosphorylations as well as STAT3 nuclear translocation. CuE could significantly inhibit the JAK2/STAT3 pathway, which makes it a desirable lead compound.

## 4. Materials and Methods 

### 4.1. Chemicals and Reagents

CuE (C_32_H_44_O_8_) was purchased from Baoji Herbest Bio-Tech Co., Ltd. (Baoji, Shanxi, China) and the purity was determined to be 99.07% based on HPLC. Fetal bovine serum (FBS) was from PAN-Biothech (Logan, UT, USA). Antibiotics and trypsin were from Macgene (Beijing, China). Matrigel was bought from BD Bioscience (Bedford, MA, USA). Antibodies against p-JNK, JNK, p-ERK, ERK, p-p38 MAPK, p38 MAPK, p-JAK3, JAK3, p-STAT3, STAT3, Cyclin A, Cyclin B1, CDK1 and GAPDH were purchased from Cell Signaling Technology (Beverly, MA, USA). Rhodamine-conjugated phalloidin was obtained from Thermo Fisher Scientific (Waltham, MA, USA). De-ionized water was obtained from Milli-Q system (Millipore, MA, USA).

### 4.2. Cell lines and Culture

Human hepatoma Huh7 and HUVEC cells were obtained from Peking Union Medical College, Cell Bank (Beijing, China). Cells were routinely maintained in high glucose DMEM supplemented with 10% FBS, 100 U/mL penicillin, and 100 μg/mL streptomycin at 37 °C in a 5% CO_2_ humidified incubator.

### 4.3. Wound Healing Assay

The inhibition effect of CuE on cell migration was assessed by wound healing assay. Huh7 cells were seeded into 6-well plates (1 × 10^6^ cells/well) and cultured until confluent. The monolayer cells were scratched with the end of 200 μL pipette tip and the plates were washed with PBS to remove the floating cells. Cells were treated with indicated concentrations of CuE for 24 and 48 h, and observed under a phase-contrast microscope. The wound area was measured by ImageJ software (ver. 1.48) and then was calculated according to the following equation: migration rate (%) = [test wound area (treated with CuE)/control area (treated with DMSO)] × 100%.

### 4.4. Transwell Migration Assay

Cell invasion was performed by transwell chambers with 8 μm pore size (Corning, Tewksbury, MA, USA). Briefly, 5 × 10^4^ cells in serum-free medium (200 μL) were seeded into upper chambers with various concentrations of CuE (0, 10, 20, and 40 nM). Then, the lower chamber was supplemented with 500 μL of DMEM medium containing 10% FBS as a chemoattractant. After incubation at 37 °C for 24 and 48 h, cotton swab was applied to remove the non-migrating cells on the upper side of the membrane and migrated cells were fixed with 90% ethanol for 30 min. Finally, cells were stained with crystal violet solution and observed under microscope and photographed. Cell numbers in three random fields were counted to evaluate transwell migration across the membrane.

### 4.5. Tube Formation Assay

HUVEC cells were seeded at a density of 5 × 10^3^ cells/well onto 96-well plates pre-coated with 100 μL of matrigel (10 mg/mL). After incubation for 6 h with or without CuE, the number of formed tubes was counted and quantified in three non-overlapping fields under a phase-contrast microscope. Tube lengths were measured in the captured images using ImageJ (ver. 1.48).

### 4.6. Chorioallantoic Membranes Assay (CAM)

Fertilized chicken eggs were incubated in a humidified atmosphere at 37 °C for five days. Then, a window (1 cm^2^) was opened aseptically on each egg shell to expose CAM. Further, coverslips containing CuE (0, 10, 20, and 40 nM) was put on CAM. After treatment for 72 h, the coverslips were removed and CAM vasculatures were photographed. Angiogenic response was evaluated by counting vessel density using ImageJ (ver. 1.48).

### 4.7. Cell Cycle Distribution Analysis

Cells were plated into 6-cell plates at a density of 2 × 10^5^ cells/well and treated with CuE (40 nM) for indicated hours. Then, cells were fixed in ice-cold ethanol (70%) at 4 °C overnight and suspended in PBS containing 0.1% Triton X-100 and 100 μg/mL RNase A. After that, cells were incubated in 5 μL of PI solution for 30 min and analyzed by flow cytometer FACS Verse (BD Biosciences, San Jose, CA, USA). Data were analyzed using Flow Jo Software (BD Biosciences, San Jose, CA, USA).

### 4.8. Rhodamine-Phalloidin Staining and Fluorescence Microscopy

Cells were seeded onto glass coverslips at a density of 1 × 10^5^ cells/well in 24-well plates. Subsequently, cells were treated with CuE (0, 10, 20, and 40 nM) for 6 h, and fixed in 4% paraformaldehyde for 30 min. After washing with PBS for 3 times, cells were permeabilized with 0.1% Triton X-100 for 30 min, blocked with 5% BSA for 30 min at room temperature, and stained with rhodamine phalloidin staining. Then, cells were counterstained with DAPI for 30 min and examined using TCS SP8 MP FLIM confocal laser scanning microscope (Leica, Wetzlar, Germany).

### 4.9. Immunofluorescent Analysis

Cells were seeded onto glass coverslips and then treated with CuE as indicated concentrations for 6 h, fixed in 4% paraformaldehyde for 30 min, and then permeabilized with 0.1% Triton X-100 for 30 min. After washing with PBS, cells were blocked with 5% BSA for 1 h at room temperature and probed with primary antibodies overnight at 4 °C. Cells were incubated with secondary antibody conjugated to rabbit Dylight-594 for 2 h at room temperature. Images were captured (594/618 nm for rabbit Dylight-594) using a confocal laser scanning microscope (TCS SP8 MP FLIM, Leica, Germany).

### 4.10. Western Blot Analysis

After treatment with indicated concentrations of CuE, cells were collected and homogenized in RIPA buffer (1×) for 30 min to provide the whole cell proteins. Protein concentrations were measured by BCA Protein Assay Kit. After 8–12% SDS-PAGE gels separation, proteins were transferred onto the PVDF membrane. Membranes were incubated with primary antibodies (1:1000) at 4 °C overnight after being blocked by 5% skimmed milk solution. Subsequently, after incubating with HRP-conjugated anti-rabbit or anti-mouse IgG secondary antibody, protein bands were developed with enhanced chemiluminescence (ECL) substrate and visualized by Tanon 5200 Imaging Analysis System (Tanon, Shanghai, China). Relative protein levels were performed by densitometry analysis using ImageJ (ver. 1.48).

### 4.11. RNA Sequencing Assay

#### Differentially Expressed Genes (DEGs) Library Construction and Sequencing

The mRNA-seq assay was conducted by Novogene (Beijing, China) with three biological replicates. DEG library was constructed for sequencing according to Illumina protocols. Briefly, total RNAs from Huh7 cells with or without 40 nM CuE were extracted by TRIzol reagent (Invitrogen, Waltham, MA, USA) and then purified using poly-T oligo-attached magnetic beads according to the standard protocol. Double-stranded complementary DNAs were synthesized by Superscript II reverse transcriptase (Invitrogen). The cDNA fragments of preferentially 150–200 bp were selected by the AMPure XP system (Beckman Coulter, Beverly, KY, USA). Clustering and sequencing were performed on a cBot Cluster Generaton System (Illumina) and Hiseq 2000 platform, respectively.

#### Functional Classification of DEGs

Differential expression analysis of two groups was performed using the DESeq R package (1.18.0). Genes with an adjusted p-value < 0.05 and log_2_ (foldchange) > 1 were assigned as differentially expressed.

Gene Ontology (GO) enrichment of DEGs was performed by Database for Annotation, Visualization and Integrated Discovery (DAVID), including CC, molecular function, and BP. Signaling pathway enrichment analysis was performed using Kyoto Encyclopedia of Genes and Genomes (KEGG) from ClueGO program, a plug-in Cytoscape software (v.3.5.1, University of California, San Diego, CA, USA).

#### Statistical Analysis

All experiments were performed at least three times with triplicate. Data were expressed as mean ± SD. Statistical comparisons were performed using GraphPad Prism 6.0 software (GraphPad Software, San Diego, CA, USA). Mean values were compared by one-way analysis of variance (ANOVA). Values of p < 0.05 were considered as statistically significant.

## 5. Conclusions

In summary, our studies demonstrate that CuE has anti-HCC effects in Huh7 cells by regulating multiple pharmacological targets including MAPKs and JAK/STAT3. Our findings strongly suggest that CuE is a multi-targeting and multi-functional anti-cancer candidate for HCC therapeutics in the clinical setting.
